# Reduced inflammation and cytokine production in NKLAM deficient mice during *Streptococcus pneumoniae* infection

**DOI:** 10.1371/journal.pone.0194202

**Published:** 2018-03-08

**Authors:** Donald W. Lawrence, Jacki Kornbluth

**Affiliations:** 1 Department of Pathology, Saint Louis University School of Medicine, St. Louis, MO, United States of America; 2 VA St. Louis Health Care System, St. Louis, MO, United States of America; Louisiana State University, UNITED STATES

## Abstract

*Streptococcus pneumoniae* is a leading cause of pneumonia and a significant economic burden. Antibiotic-resistant *S*. *pneumoniae* has become more prevalent in recent years and many pneumonia cases are caused by *S*. *pneumoniae* that is resistant to at least one antibiotic. The ubiquitin ligase natural killer lytic-associated molecule (NKLAM/RNF19b) plays a role in innate immunity and studies using NKLAM-knockout (NKLAM-KO) macrophages have demonstrated that NKLAM positively affects the transcriptional activity of STAT1. Using an inhalation infection model, we found that NKLAM-KO mice had a significantly higher lung bacterial load than WT mice but had less lung inflammation. Coincidently, NKLAM-KO mice had fewer neutrophils and NK cells in their lungs. NKLAM-KO mice also expressed less iNOS in their lungs as well as less MCP-1, MIP1α, TNFα, IL-12, and IFNγ. Both neutrophils and macrophages from NKLAM-KO mice were defective in killing *S*. *pneumoniae* as compared to wild type cells (WT). The phosphorylation of STAT1 and STAT3 in NKLAM-KO lungs was lower than in WT lungs at 24 hours post-infection. NKLAM-KO mice were afforded some protection against a lethal dose of *S*. *pneumoniae* compared to WT mice. In summary, our novel data demonstrate a role for E3 ubiquitin ligase NKLAM in modulating innate immunity via the positive regulation of inflammatory cytokine expression and bactericidal activity.

## Introduction

*Streptococcus pneumoniae* is a Gram-positive bacterium and a significant cause of human infection. *S*. *pneumoniae* is a common causative agent of bacterial pneumonia, meningitis, and bacteremia cases, and is a significant etiological factor in the morbidity and mortality of children, the elderly, and patients with defective immune systems [1]. Administration of antibiotics is the standard treatment; however, antibiotic-resistant *S*. *pneumoniae* is becoming more prevalent [2]. There are 1.2 million new cases of *S*. *pneumoniae* infections each year and an increasing number of pneumonia cases are caused by *S*. *pneumoniae* that is resistant to at least one antibiotic [1]. Additionally, *S*. *pneumoniae* infections are associated with significant financial burden. Nearly $100 million per year is spent treating pneumococcal disease [3]. Thus, research into non-antibiotic therapies to combat drug resistant *S*. *pneumoniae* is necessary.

Natural killer lytic-associated molecule (NKLAM) is a membrane-bound ubiquitin ligase and member of the ring in between ring (RBR) family of proteins. Currently there are 15 members of the family [4], the most well-studied member being Parkin. In macrophages, NKLAM expression is upregulated in response to cytokines (e.g. interferon gamma; IFNγ), bacteria and bacterial products such as lipopolysaccharide (LPS) [5]. Studies from our laboratory have shown that NKLAM is localized to the phagosome and ubiquitinates phagosomal proteins [5]. Additionally we have demonstrated that bone marrow-derived and peritoneal macrophages from NKLAM-knockout (KO) mice have a significantly defective killing response against *Escherichia coli*, suggesting that NKLAM is an important component of the innate immune system [5]. Although the role of RBR ubiquitin ligases in the pathophysiology of infectious disease is not well-studied, there is evidence that RBR ligases play a significant role in host defense [4]. For example, Parkin has been shown to be involved in host resistance against *Mycobacterium tuberculosis* [6] and variants in the regulatory region of the Parkin gene are risk factors for leprosy [7].

More recent research from our laboratory demonstrated that NKLAM associates with STAT1 and mediates its K63-linked ubiquitination [8]. We have also shown that NKLAM plays significant role in facilitating the binding of STAT1 to consensus binding sites (GAS; gamma activated sites) within the promoter region of iNOS. Without NKLAM, IFNγ-stimulated NKLAM-KO macrophages express significantly less iNOS than wild type (WT) macrophages [8]. In response to bacteria or bacterial products, STAT1 is phosphorylated on tyrosine 701, dimerizes and translocates to the nucleus where it is involved in the transcription of many immunologically important genes. Mutations in the STAT1 gene are associated with increased susceptibility to pathogens thus strengthening the importance of STAT1 as a vital component of host defense [9–11]. Investigation into the mechanisms of how NKLAM regulates STAT protein function may provide additional targets for therapeutic control of inflammation.

There is an emerging body of evidence suggesting that RBR ubiquitin ligases are involved in regulating the expression of pro-inflammatory cytokines. Studies from our laboratory have shown that NKLAM is involved in enhancing the expression of IFNγ or RANTES/CCL5 in response to LPS and IFNγ, respectively [8, 12]. The knockdown of E3 ubiquitin ligase Parkin with siRNA or the use of Parkin-KO mice demonstrated that Parkin, in part, regulates the expression of lung IL-6 and TNFα as well as the expression of IL-6 and IL-8 from endothelial cells [13]. Additionally, down-regulation of Parkin expression in THP-1, monocyte-derived macrophages and Schwann cells was associated with decreased IL-6 and MCP-1/CCL2 expression in response to LPS or mycobacteria [14]. Conversely, Inn et al. showed that the deletion of RBR ubiquitin ligases HOIL-1L and HOIP resulted in enhanced IFNβ production in response to Sendai virus infection [15]. Collectively, these observations provide evidence that RBR ubiquitin ligases play an integral role in cytokine expression; however, their precise immunological function may differ depending on cell type or infectious organism.

In this present study, we used a *S*. *pneumoniae* pneumonia model and show that NKLAM-KO mice are less able to kill these bacteria *in vitro* and *in vivo*. This defect correlated with lower levels of inflammatory chemokines that are necessary for leukocyte recruitment to sites of inflammation. Our present study presents novel data that support a role for NKLAM as a component of the innate immune system and a positive regulator of inflammatory cytokine expression.

## Materials and methods

### Ethics statement

All mouse experiments were reviewed and approved by the Institutional Animal Care and Use Committee at Saint Louis University (Protocol#1287 and 2672) and the Saint Louis Veterans Affairs Healthcare System (Protocol#1108–3115 and 1701–522). This study was carried out in strict accordance with the recommendations in the Guide for the Care and Use of Laboratory Animals of the National Institutes of Health. For *S*. *pneumoniae* infection, mice were anesthetized with an intraperitoneal injection of xylazine/ketamine. Animals were euthanized with carbon dioxide.

### NKLAM knockout mouse generation

The generation of mice deficient in NKLAM was performed in our laboratory [16]. Briefly, 129Sv/Ev (inGenious Targeting Laboratory) embryonic stem cells containing the NKLAM targeting construct were microinjected into B6 blastocysts. This construct generates deletion of exons 2–5 of NKLAM, which encode the RBR domains and most of the transmembrane domains. Chimeric males were mated with B6 mice and the progeny were screened for germline transmission of the NKLAM KO allele. Heterozygotes were crossed to obtain homozygous offspring. Mice were backcrossed for 11 generations onto a B6 background [16]. We simultaneously maintain lines of both NKLAM KO and B6 mice for experiments.

### Mouse pneumonia model

*Streptococcus pneumoniae* (ATCC #6301; serotype 1) were grown on blood agar plates overnight at 37°C and aliquots of 1 x 10^8^ bacteria were recovered as determined by spectrophotometry. The bacteria were washed once in ice-cold PBS and resuspended in PBS at a concentration of 10 x 10^6^ CFU/30 μL. The bacteria were kept on ice until needed. Mice were anesthetized by intraperitoneal injection (100 μL/10g body weight) of xylazine (1mg/mL) and ketamine (7.5 mg/mL) and 30 μL of bacteria culture or sterile PBS was administered to the nares of each mouse. Following infection, the mice were monitored for signs of distress. At 24 and 48h-post infection, the mice were euthanized with CO_2_. The lungs were removed and homogenized for 30 sec in sterile PBS (1 mL) using a tissuemiser (Fisher Scientific). For the survival experiments, WT and NKLAM-KO mice were infected nasally with 5 x 10^7^ *S*. *pneumoniae* CFU and monitored several times a day for signs of severe sickness (e.g. lethargy, hypothermia, piloerection, inability to maintain upright posture). The total duration of the experiment was 120 hours. Extremely ill mice were euthanized immediately by CO_2_ asphyxiation and scored dead for the purposes of the experiment.

### Bone marrow-derived neutrophil and macrophage isolation

Wild type C57BL/6 and corresponding age-matched NKLAM-KO mice were used in all studies. Both males and females were used. Neutrophils were isolated according to Swamydas et al. [17]. Briefly, euthanized mice were sprayed with 70% ethanol and the femurs and tibias were dissected. The bones were flushed through a 70 μm mesh filter with Hank’s Balanced Salts Solution (HBSS) supplemented with 10% FBS and 2 mM EDTA. The collected cells were pelleted and the red cells were lysed by resuspending the cell pellet in 20 mL of 0.2% NaCl for 20 sec followed by 20 mL of 1.6% NaCl. After pelleting, the cells were washed twice in HBSS plus 10% FBS without EDTA. The cell pellet was resuspended in 1 mL of PBS, layered over a discontinuous Histopaque gradient and centrifuged for 30 min at 2000 x g. The bone marrow-derived neutrophils were collected at the interface of the Histopaque 1119 and Histopaque 1077 layers. The collected neutrophils were washed once in HBSS, counted and the viability was determined with trypan blue. Cytospins were performed to verify the neutrophil population.

For isolation of bone marrow-derived macrophages, euthanized mice were sprayed with 70% ethanol and the femurs and tibias were dissected. The bones were flushed with DMEM and the collected marrow was resuspended in BM20 media (DMEM supplemented with 20% fetal bovine serum, 20% L929-cell conditioned media, 2 mM L-glutamine, 100 U/mL penicillin,100 U/ml streptomycin, and 1 mM sodium pyruvate). The bone marrow cells were cultured for 7 days in 100 mm non-tissue culture petri dishes with a partial media change on day 3.

### *S*. *pneumoniae* killing assay

WT and NKLAM-KO mice were infected with 10 x 10^6^ CFU of *S*. *pneumoniae* as described above. The lungs were harvested at 24 and 48h then homogenized in 1 mL of sterile PBS. Ten-fold serial dilutions were plated on blood agar plates for 18h at 37°C with 5% CO_2_. The number of colonies was multiplied by the dilution factor and divided by the volume (mL) of the plated diluted suspension to determine the number of bacteria per lung.

For the *in vitro* killing assays, 1 x 10^6^ bone marrow-derived neutrophils or macrophages were resuspended in 180 μL of HBSS plus 0.1% gelatin. For *S*. *pneumoniae* opsonization, 1 x 10^4^ bacteria were incubated in 20 μL of HBSS plus 0.1% gelatin plus 10% normal mouse serum for 60 min on ice. The cells and bacteria were combined and incubated at 37°C for 90 min with rotation. The cells were lysed by the addition of 1 mL of sterile water and the cultures were serial diluted 1:10 with sterile water containing 0.01% BSA then plated on blood agar plates.

### Lung morphology

Mice were euthanized by CO_2_ asphyxiation and the lungs were gently inflated with 10% formalin. Lungs were removed and after ethanol dehydration, embedded in paraffin. Lung sections were stained with hematoxylin and eosin or processed for immunofluorescence. Sections of formalin fixed, paraffin embedded lungs were deparaffinized and rehydrated followed by incubation in antigen retrieval solution (1 mM EDTA, 0.05% Tween 20) for 3 min at 120°C. The sections were blocked and incubated with 1:800 rabbit anti-pSTAT1 (Tyr701) overnight at 4°C. After washing in Tris-buffered saline (TBS) the sections were incubated with anti-rabbit HRP for 60 min followed by amplification with alexafluor-594 tyramide (Invitrogen). For the myeloperoxidase (MPO) staining, lung sections were treated with 0.3% H_2_O_2_, blocked and incubated with 1:100 anti-MPO (Abcam #ab9535). After washing in PBS, the sections were incubated with anti-rabbit HRP for 60 min, then visualized with 3,3′-diaminobenzidine, and then counterstained with hematoxylin.

### Immunoblotting

Mouse lung proteins were separated using SDS-PAGE then transferred to PVDF membrane. Membranes were blocked with 1% (wt/vol) BSA in TBS plus 0.1% Tween-20 (TBS-T) then incubated in primary antibody (1:1000 dilution) with rocking overnight at 4°C. The rabbit monoclonal antibodies for STAT1, pSTAT1 (Tyr701), STAT3, and pSTAT3 (Tyr705) were purchased from Cell Signaling. The monoclonal beta actin antibody (clone AC-15) was from Sigma-Aldrich. The anti-NKLAM antibody has been described previously [18]. The iNOS (clone 2/iNOS) antibody was purchased from Becton Dickinson. After three washes in TBS-T, the blots were probed with HRP-conjugated secondary antibodies and the proteins were visualized with BioRad Immun-Star Western C chemiluminescence kit. Images were captured and analyzed using a BioRad Chemidoc XRS+ imager.

### In gel phosphatase assay

Aliquots (50 μg) of non-reduced, non-denatured lung homogenate were separated by native-PAGE using a 7.5% gel. The gel was washed 3 times for 10 min each in deionized water. The gels were then incubated in reaction buffer containing: 1 M Tris, pH 7.5, 10% Tween 20, 1M DTT, 1M MnCl_2_, and 500 mM 4-methylumbelliferyl phosphate for 20 min at 37°C. Phosphatase activity was visualized using the ethidium bromide filter set in a BioRad Chemidoc XRS+ imager. ImageLab software V5.0 (BioRad) was used to ensure band intensities were within the linear range.

### Quantitative PCR

Fifty microliters of lung homogenate were added to 350 μL of RLT Plus buffer (Qiagen) and the total RNA was isolated using a Qiagen RNeasy kit per manufacturer’s instructions. One hundred nanograms of RNA was used to synthesize cDNA using the Taqman reverse transcription kit (Applied Biosystems). Quantitative PCR was performed in 96-well plates with iTaq Universal SYBR Green Supermix (Bio-Rad) or Taqman Gene Expression Master Mix (Applied Biosystems) and primer probes. 18S served as an internal standard for normalization. For graphical presentation of quantified RT-PCR results, the mean ΔΔCT values for each gene were calculated by subtracting the mean ΔCT value of the reference gene (18S) from the mean ΔCT value of the target gene. Data were calculated as 2^ΔΔCT^ with the PBS control values defined as 1.

### Cytokine profile using cytometric bead array

Cytokines (IL-6, IL-10, IL-12 p70, TNFα, MCP-1, and IFNγ) were measured in lung homogenate or plasma by cytometric bead array using the mouse inflammation kit (Mouse Inflammation Kit, catalog# 552364, BD Biosciences). For plasma collection, blood was drawn into EDTA-coated syringes via cardiac puncture then centrifuged for 10 min at 4000 x g. The plasma and lung homogenate samples were briefly treated with 1% paraformaldehyde to kill any remaining *S*. *pneumoniae* just prior to cytokine concentration determination by flow cytometry using a Becton-Dickinson FACS Canto II cytometer.

### Flow cytometry

Infected mouse lungs were mechanically homogenized using the gentleMACS tissue dissociator (Miltenyi). The resulting cell suspension was passed through a 70 μm mesh filter to remove cell clumps then washed once in ice-cold PBS. Cells (500,000) were incubated with Fc block for 20 min on ice then stained with anti-mouse CD45-PerCP, F4/80-efluor 450, CD11b-PE, NK1.1-APC, Ly-6G (Gr-1)-FITC, and CD3-Alexa Fluor 700 for 30 min on ice in the dark. Cells were washed once in ice-cold staining buffer then fixed with 1% formaldehyde. Flow cytometric data were analyzed with FlowJo software (Treestar, Ashland, OR).

### Statistical analysis

Statistical differences were assessed using a two-tailed, unpaired Student’s *t*-test, a non-parametric Mann-Whitney test or log rank test for significance with Microsoft Excel software or GraphPad software. A p value of 0.05 or less was considered statistically significant.

## Results

### Reduced *in vivo* and *in vitro* clearance of *S*. *pneumoniae* by NKLAM-KO mice

**Fig 1:**
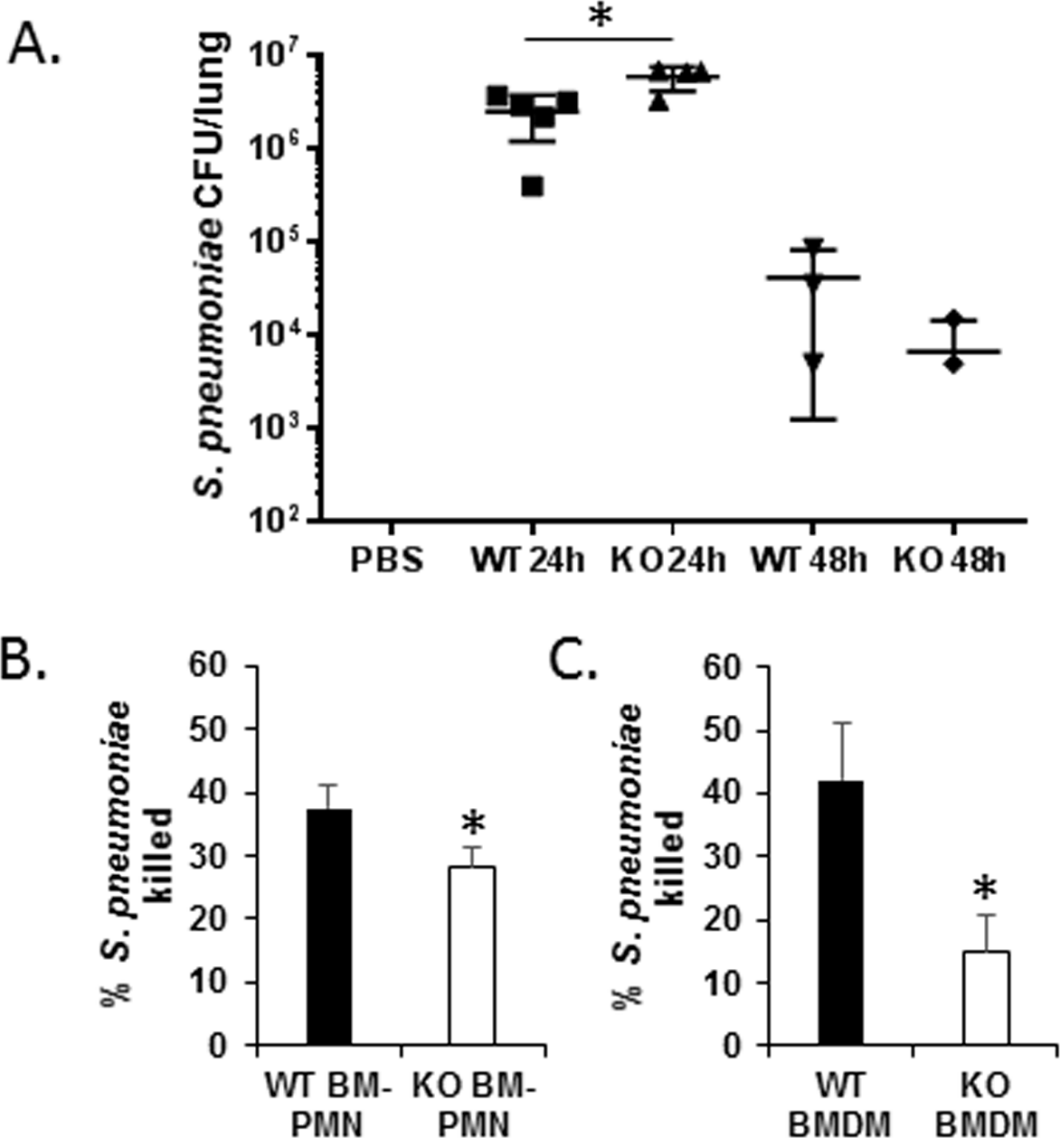
The absence of NKLAM impairs *S*. *pneumoniae* killing. (A) WT and NKLAM-KO mice were infected by nasal administration of 10 x 10^6^ CFU of *S*. *pneumoniae*. The lungs were harvested at 24 or 48h, homogenized in sterile PBS and an aliquot was plated on blood agar plates to determine bacterial load. Instillation of sterile PBS was used as a control. Data represent mean ± SD of 1 of 3 independent experiments. (n = 8–11 total mice for each condition); * p < 0.05. (B) Bone marrow-derived neutrophils (BM-PMN) or C) macrophages (BMDM) were incubated with serum-opsonized *S*. *pneumoniae* at an MOI of 0.01. The percentage *S*. *pneumoniae* killed was determined relative to control cultures lacking neutrophils or macrophages. n = 3; p < 0.05.

To assess the role of NKLAM in *S*. *pneumoniae* clearance, WT and NKLAM-KO mice were intranasally infected with 10 x 10^6^ colony forming units (CFU) of *S*. *pneumoniae* and the lung bacterial load was determined after 24 and 48h of infection. As shown in Fig 1, the average number of *S*. *pneumoniae* CFU/lung in WT mice after 24h of infection was 2.5 x 10^6^. In contrast, on average the lungs of NKLAM-KO mice contained 6 x 10^6^ CFU of *S*. *pneumoniae*, a 2.4-fold difference. By 48h there were no significant differences in *S*. *pneumoniae* CFU/lung between the genotypes. To assess the killing ability of leukocytes key to fighting infection, we used bone marrow-derived neutrophils (BM-PMN) and macrophages (BMDM) in *S*. *pneumoniae* killing assays. As show in Fig 1B and 1C, both neutrophil and macrophages from NKLAM-KO mice are significantly deficient in killing *S*. *pneumoniae* compared to WT cells.

### Leukocyte recruitment into the lung is greater in WT than NKLAM-KO mice

**Fig 2:**
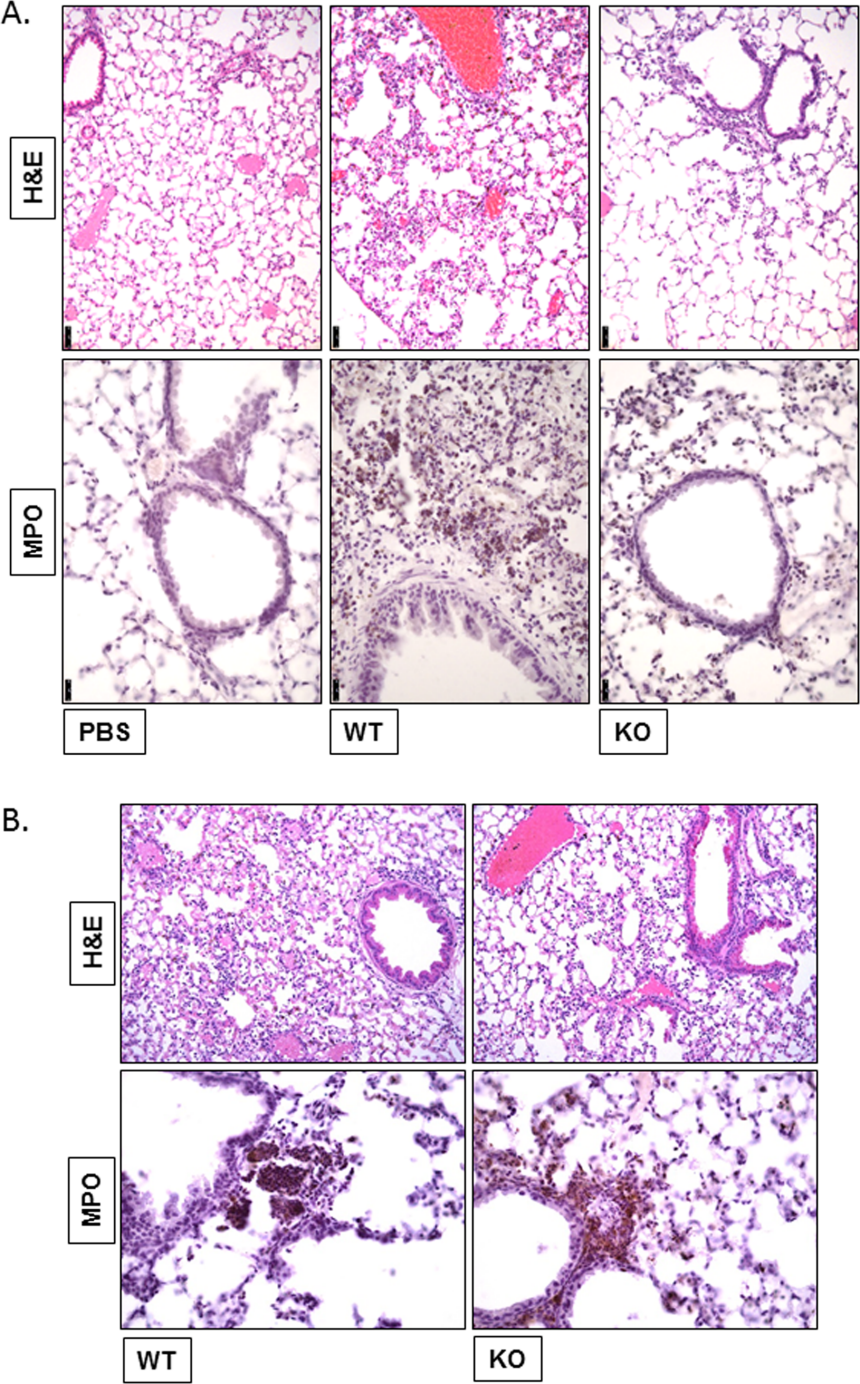
Lack of NKLAM is associated with reduced lung inflammation. (A) Lungs from 24h-infected or (B) 48h-infected *S*. *pneumoniae*-infected (10 x 10^6^ CFU/mouse) WT and NKLAM-KO or PBS-treated mice were formalin-fixed, embedded in paraffin and sections were stained with H&E or anti-mouse MPO. Magnification, x 200 for H&E, x 400 for MPO. Black bar equals 50 μm. Data are representative of two experiments, with n = 2 mice per group per experiment.

Leukocyte infiltration into the lung is a key characteristic of pneumococcal infection. We performed hematoxylin and eosin (H&E) and anti-myeloperoxidase (MPO) staining of formalin fixed, paraffin-embedded lung sections to assess the degree of leukocyte infiltration during *S*. *pneumoniae* infection. In the absence of *S*. *pneumoniae*, the mice did not exhibit any lung pathology (PBS control). After 24h of *S*. *pneumoniae* infection, lungs from WT mice showed a considerable amount of leukocyte infiltration, significant alveolitis and hemorrhage. In contrast, the lungs from infected NKLAM-KO mice had less severe lung pathology and less leukocyte infiltration (Fig 2A). At 48h-post infection, the extent of inflammation as depicted by H&E and MPO staining was similar between WT and NKLAM-KO mice (Fig 2B).

### Neutrophil and NK cell lung infiltration by flow cytometry and neutrophil function

**Fig 3:**
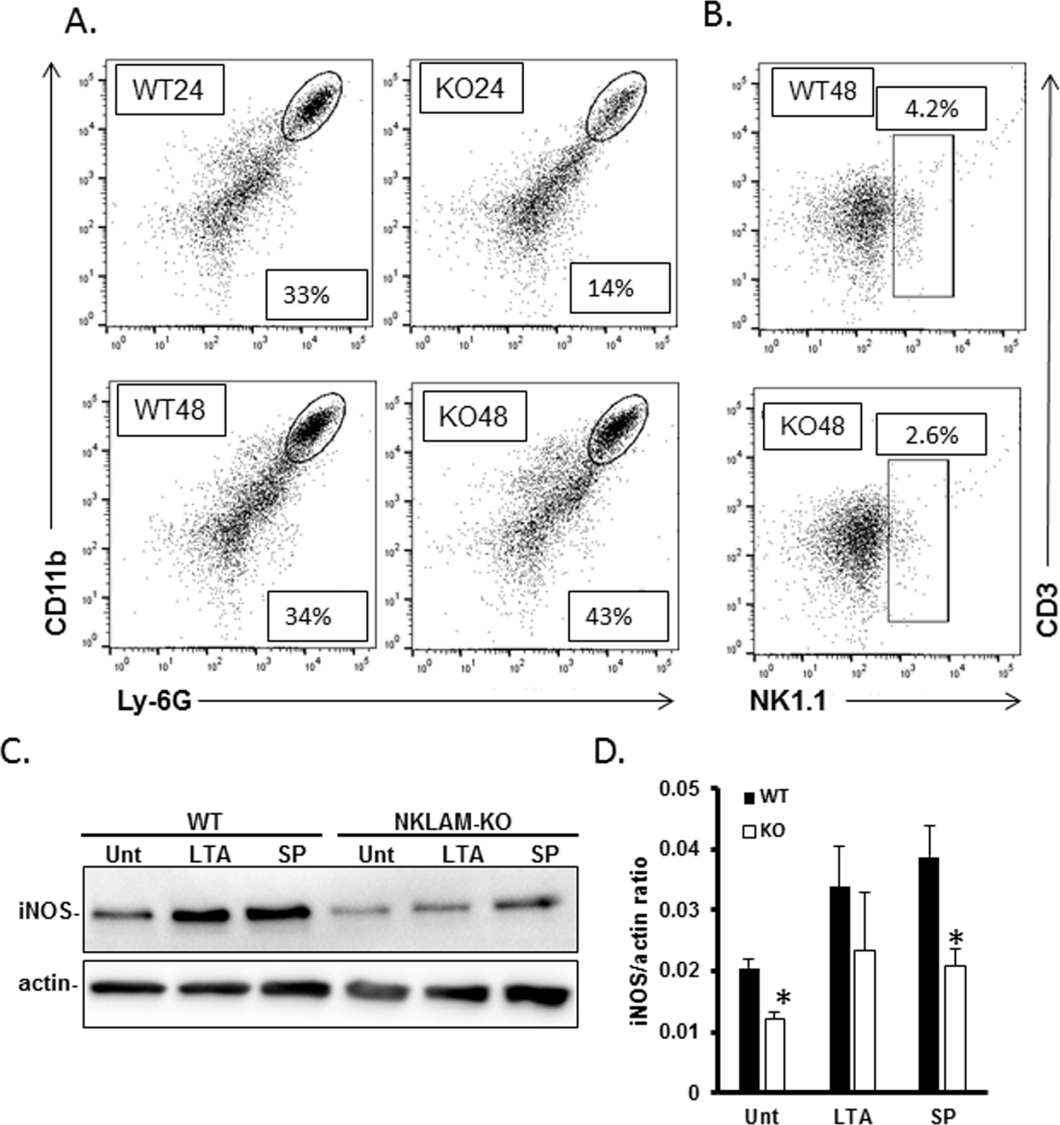
Neutrophil and NK cell lung infiltration and bone marrow-derived macrophage iNOS expression. Cells were isolated from infected lungs at 24 and 48h post infection and stained for CD45, CD3, NK1.1, CD11b and Ly-6G. CD45^+^ cells were gated and the percentage of CD11b^+^/Ly-6G^+^ (A) or CD3^-^/NK1.1^+^ (B) cells within the CD45^+^ population was determined. A representative histogram is shown for each group (n = 3–4 mice per group). (C) BMDM were treated with 100 μg/mL lipoteichoic acid (LTA) or formalin-fixed *S*. *pneumoniae* (SP) at an MOI of 10 for 18 hr at 37°C and protein lysates were immunoblotted for iNOS protein. Beta actin was used as a loading control. Immunoblots represent 1 of 3 identical experiments, (D) Graphical representation of C. n = 3; *p < 0.05.

By H&E staining we observed significant leukocyte infiltration into the lung. This subsequent series of experiments was designed to determine which leukocyte subsets were present in mouse lungs in response to *S*. *pneumoniae* infection. A single cell suspension from NKLAM-KO and WT infected lungs was labeled with anti-CD45 (hematopoetic cell marker) along with the macrophage marker F4/80, CD11b (leukocyte marker), Ly-6G, which is expressed on neutrophils, NK1.1 (NK cell marker) and CD3 (T cell marker). We gated on the CD45^+^ population then examined each subpopulation. Representative histograms are shown in Fig 3A and 3B. Neutrophils (CD11b^+^/Ly-6G^+^) made up 33% (3.3 ± 0.75 x 10^5^ cells/10^6^ CD45^+^ cells) of the CD45 positive cells in the lungs of WT mice 24h after *S*. *pneumoniae* infection. In contrast, NKLAM-KO mice showed evidence of a significant attenuation in neutrophil migration into the lung as the CD11b^+^/Ly-6G^+^ population was 14% (1.4 ± 0.74 x 10^5^ cells/10^6^ CD45^+^ cells) of the CD45^+^ population; p < 0.05. By 48h-post infection, the neutrophil populations were similar in WT and NKLAM-KO mouse lungs (WT: 3.4 ± 0.14 x 10^5^ cells/10^6^ CD45^+^; KO: 4.3 ± 0.81 x 10^5^ cells/10^6^ CD45^+^ cells). Additionally, the proportion of NK1.1^+^/CD3^-^ cells (NK population) was higher in WT lungs than in NKLAM-KO lungs at 48h post-infection WT 4.2% (4.2 ± 0.28 x 10^4^ cells/10^6^ CD45+ cells); KO 2.6% (2.6 ± 0.68 x 10^4^ cells/10^6^ CD45^+^ cells); p ≤ 0.05. There were no significant differences in the numbers of cells in the lung homogenates of each cell type (WT 24h, 5.0 ± 2.4 x 10^7^; NKLAM-KO 24h, 3 ± 0.83 x 10^7^; WT 48h, 5.5 ± 2.3 x 10^7^; NKLAM-KO 48h, 4.2 ± 0.18 x 10^7^).

Previous research from our laboratory demonstrated that macrophages from NKLAM-KO mice are defective in mounting effective immune responses against *E*. *coli* [5]. Our next series of experiments was designed to examine immune function in WT and NKLAM-KO BMDM. We found that macrophage iNOS expression in response to Gram-positive Toll-like receptor agonist, lipoteichoic acid (LTA) or formalin-fixed *S*. *pneumoniae* (SP) was significantly less in NKLAM-KO macrophages as compared to WT macrophages (Fig 3C and 3D). This observation is in line with our previous results that show NKLAM-KO BMDM express less iNOS in response to LPS than WT macrophages [12] and suggests a more global role for NKLAM in mediating iNOS expression. Collectively, these data show that NKLAM-KO mice experience a significant defect in leukocyte transmigration into the lung in the response to *S*. *pneumoniae* and that macrophages from NKLAM-KO mice have a defect in a key bactericidal mechanism.

### NKLAM-KO mice express less lung inflammatory cytokines in response to *S*. *pneumoniae* infection

**Fig 4:**
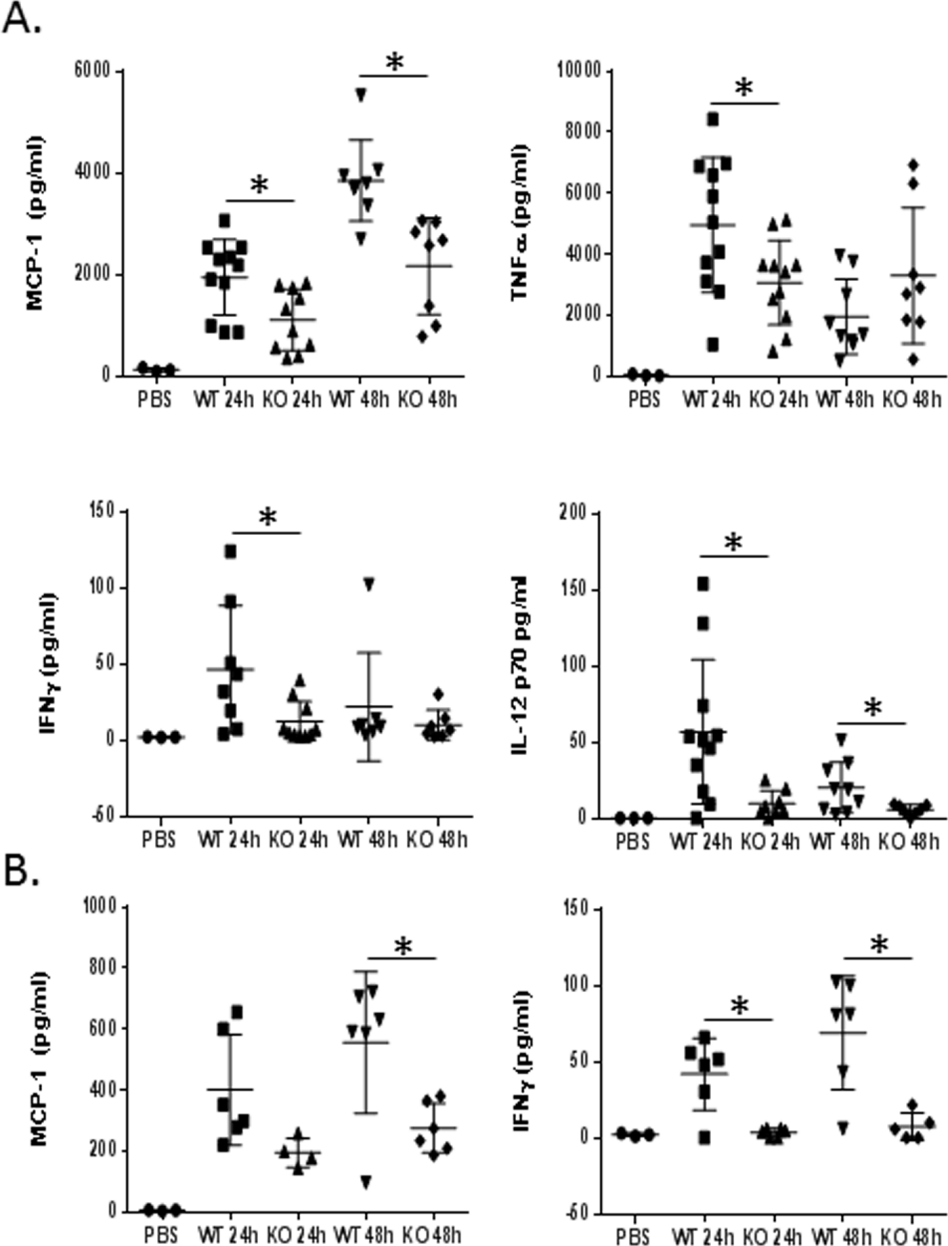
Mouse inflammatory cytokine profile from *S*. *pneumoniae*-infected WT and NKLAM-KO mice. Mouse inflammatory cytokines (MCP-1, TNΦα, IFNγ and IL-12p70) were evaluated in lung homogenates (A) and plasma (B) from infected (24 and 48hr) WT and NKLAM-KO mice by cytometric bead array. Data shown were pooled from 3 independent experiments (n = 8–11 mice per group). * p ≤ 0.05.

Lung infection with *S*. *pneumoniae* induces the expression of inflammatory cytokines within the lung. We used lung homogenates and plasma to assess the concentration of six inflammatory cytokines in infected WT and NKLAM-KO mice lungs. At 24h-post infection (Fig 4A) the concentration of MCP-1, TNFα, IFNγ and IL-12p70 and were all significantly reduced in NKLAM-KO lungs compared to WT lungs. The levels of MCP-1 and IL-12p70 in NKLAM-KO lungs were also significantly reduced at 48h-post infection. In the plasma (Fig 4B), NKLAM-KO mice had significantly reduced levels of MCP-1 at 48h post-infection. The levels of IFNγ in plasma from NKLAM-KO mice were reduced at 24 and 48h post-infection in comparison to WT mice.

### NKLAM-KO mice have reduced expression of STAT1-regulated genes in their lungs in response to *S*. *pneumoniae*

**Fig 5:**
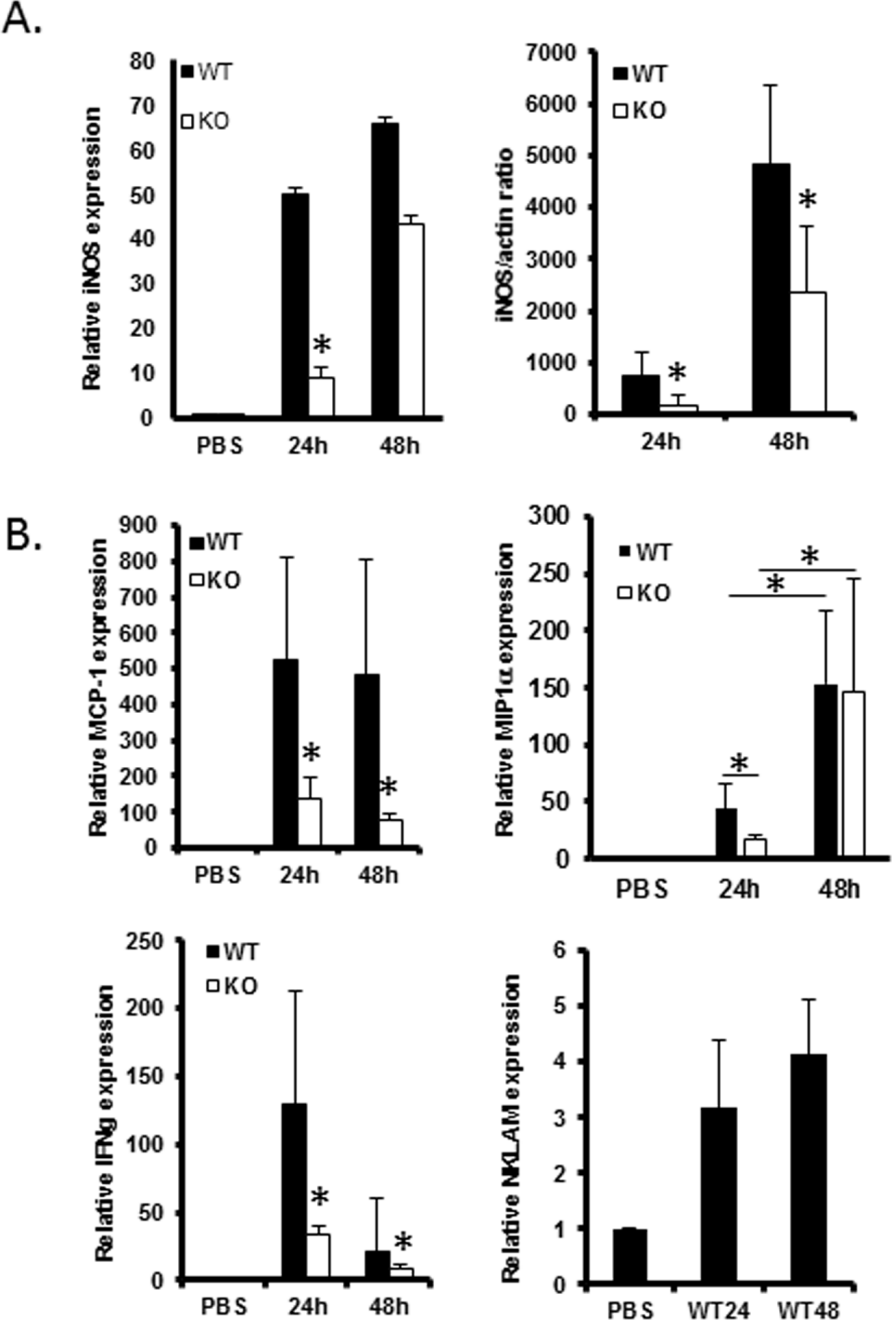
Expression of STAT1-regulated genes. (A) mRNA isolated from the lungs of infected WT and NKLAM-KO mice was used to determine the relative expression of iNOS. Lung homogenates were used to assess iNOS protein expression. Beta actin was used as a loading control. (B) Quantitative PCR was used to determine the relative expression of MCP-1, MIP1α, IFNγ and NKLAM in infected lungs. The mRNA levels (mean ± SD) are expressed relative to PBS-treated mice. * p < 0.05; n = 8–11 mice per group.

Previous studies from our laboratory have demonstrated that NKLAM affects the binding of STAT1 to GAS elements and facilitates that transcription of STAT1-regulated genes such as iNOS [8]. The expression of iNOS is elevated in response to bacteria, bacteria products as well as interferons type I and II. As shown in Fig 5A, iNOS expression, as determined by both qPCR and immunoblot analysis, is significantly lower in *S*. *pneumoniae-*infected NKLAM-KO lungs than in WT lungs. In the next set of experiments, we used qPCR to examine the expression of several genes that are known to be regulated by STAT1 or have STAT1 consensus binding sites within their promoters. As shown in Fig 5B chemokine monocyte chemotactic protein-1 (MCP-1/CCL2) was also significantly lower in NKLAM-KO lungs. Macrophage inflammatory protein 1 alpha (MIP1α/CCL3) is a chemokine produced by macrophages, monocytes, and natural killer (NK) cells [19]. MIP1α is a chemoattractant for neutrophils and monocytes [20]. The promoter for MIP1α contains consensus binding sites for STAT1 and STAT3 (DECODE database, SABiosciences). As shown in Fig 5B, MIP1α is upregulated during infection and the expression of MIP1α was lower in the lungs of *S*. *pneumoniae*-infected NKLAM-KO mice at 24h. IFNγ expression was also significantly lower in NKLAM-KO lungs than in WT lungs at 24h and 48h post-infection. Lastly, we determined the expression of NKLAM in infected WT lungs. NKLAM expression was significantly induced by *S*. *pneumoniae* infection. NKLAM is also a STAT1 target gene and is transcriptionally upregulated by IFNγ [21].

### Decreased STAT tyrosine phosphorylation in *S*. *pneumoniae*-infected NKLAM-KO mice

**Fig 6:**
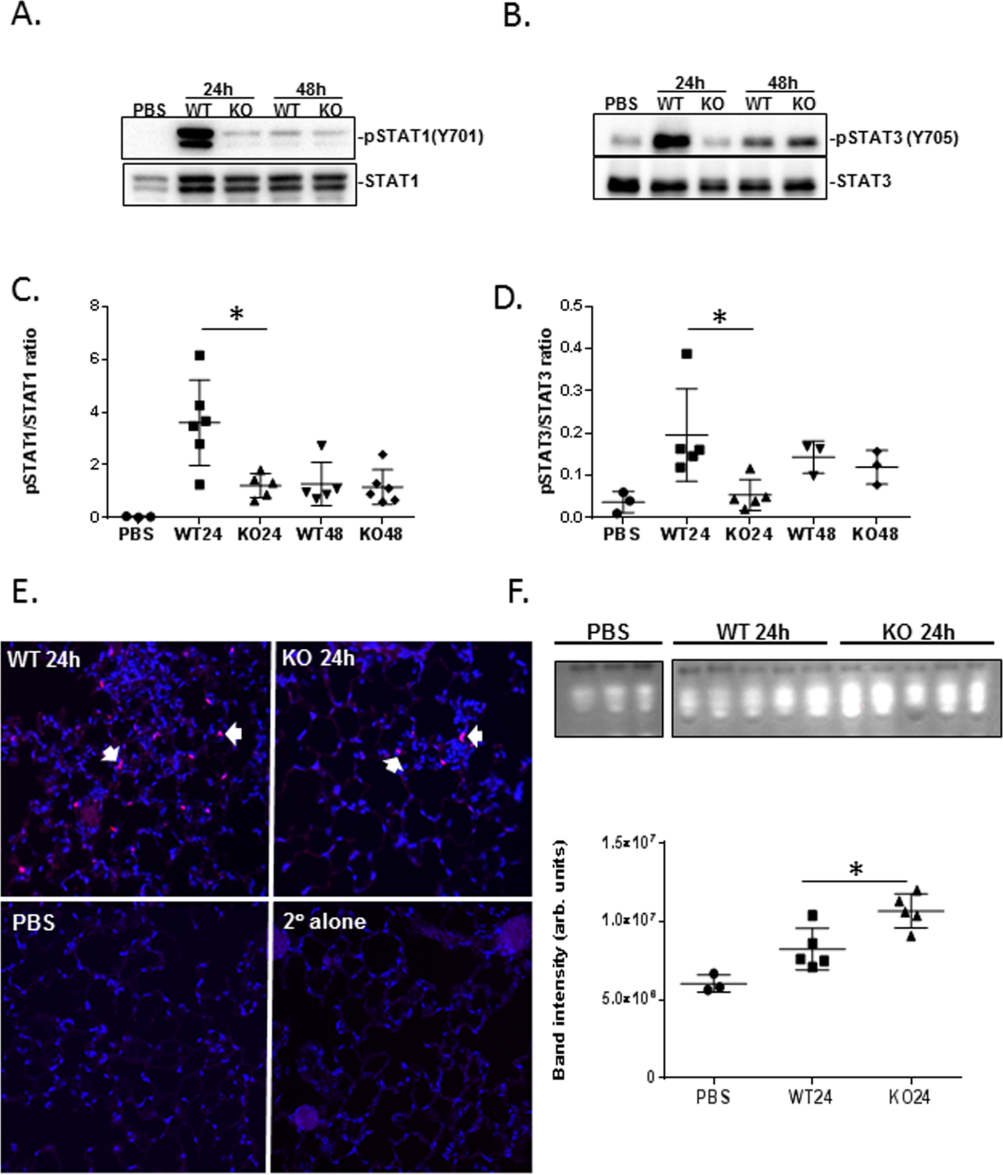
*S*. *pneumoniae*-induced phosphorylation of STAT1 and STAT3 is diminished in NKLAM-KO mice. Lung homogenates from *S*. *pneumoniae*-infected WT and NKLAM-KO mice were immunoblotted for (A) STAT1 and pSTAT1 (Tyr701) and (B) STAT3 and pSTAT3 (Tyr705). Each lane represents an individual mouse and is representative of the overall experiments. Immunoblots were used to calculate the ratio of pSTAT1 (Tyr701)/STAT1 (C) and pSTAT3 (Tyr705)/STAT3 (D). Data represent the mean ± SD of 1 of 3 representative experiments (n = 3–5 mice per group per experiment); * p ≤ 0.05. (E) Sections from paraffin-embedded lungs from *S*. *pneumoniae*-infected (24h) WT and NKLAM-KO mice were stained for pSTAT1 (Tyr701) (red) and DAPI (blue). Colocalization is depicted in pink (arrows). (n = 2–3 mice per group). (F) Aliquots (50 μg) of lung homogenate from PBS control mice and *S*. *pneumoniae*-infected (24h) WT and NKLAM-KO mice were separated by native gel electrophoresis then incubated with 4-methylumbelliferyl phosphate to visualize phosphatase activity. Each lane represents an individual mouse. Graph represents the mean band intensity ± SD. Results are representative of two experiments with 3–5 mice per group per experiment; * p ≤ 0.05.

Members of the STAT family are phosphorylated in response to bacteria and bacterial products and STAT family phosphorylation correlates with STAT-mediated gene expression. Previous studies from our laboratory have shown that NKLAM-mediated STAT1 ubiquitination has a positive effect of the ability of STAT1 to bind DNA and STAT1-mediated gene transcription is decreased in NKLAM-KO macrophages [8]. Lung homogenates were used to assess the phosphorylation state of STAT1 and STAT3 at 24 and 48h-post infection with *S*. *pneumoniae*. As shown in Fig 6A and 6C, STAT1 phosphorylation at 24h is significantly greater (2.9 fold) in the lungs of WT mice than in NKLAM-KO mice. Lung homogenates from control mice given intranasal administration of PBS showed no STAT1 phosphorylation. Similarly, at 24h post infection the levels of lung phospho-STAT3 (pSTAT3) are greater (3.6 fold) in WT mice than in NKLAM-KO mice (Fig 6B and 6D). For both genotypes, the levels of phospho-STAT1 and phospho-STAT3 are not significantly different 48h after infection. Our results agree with others that have found *S*. *pneumoniae* induces STAT1 and STAT3 phosphorylation [22]. We next assessed pSTAT1 (Tyr701) expression by immunofluorescence. Lung tissue from WT and NKLAM-KO mice infected with *S*. *pneumoniae* for 24h was stained for pSTAT1. Nuclei were counterstained with DAPI. Immunofluorescence indicates that there are more pSTAT1-positive nuclei in the lungs of WT mice expressing pSTAT1 than in the lungs of NKLAM-KO mice (Fig 6E). This is consistent with the immunoblot data showing higher levels of pSTAT1 protein in lungs of infected WT mice than in NKLAM-KO mice (Fig 6A). There are no pSTAT1-positive nuclei in PBS-treated or secondary antibody alone controls.

Protein phosphorylation state is the balance between kinase and phosphatase activity; thus, the lower levels of phosphorylation of pSTAT1 and pSTAT3 we observed in NKLAM-KO lungs (Fig 6A and 6B) could be the result of enhanced phosphatase activity. We used an in-gel phosphatase assay to assess the overall lung phosphatase activity after infection with *S*. *pneumoniae*. As the major differences in STAT phosphorylation between WT and NKLAM-KO mice were observed at 24h we assayed homogenate from mouse lungs 24h-post infection. As shown in Fig 6F, *S*. *pneumoniae* infection induced an increase in overall phosphatase activity in the lungs of WT and NKLAM-KO mice. However, the phosphatase activity was significantly higher in NKLAM-KO mouse lungs than in WT mouse lungs. This observation correlated with the lower phosphorylation state of lung STAT1 and STAT3 in NKLAM-KO mice compared to WT mice at 24h-post infection.

### *S*. *pneumoniae* survival study

**Fig 7:**
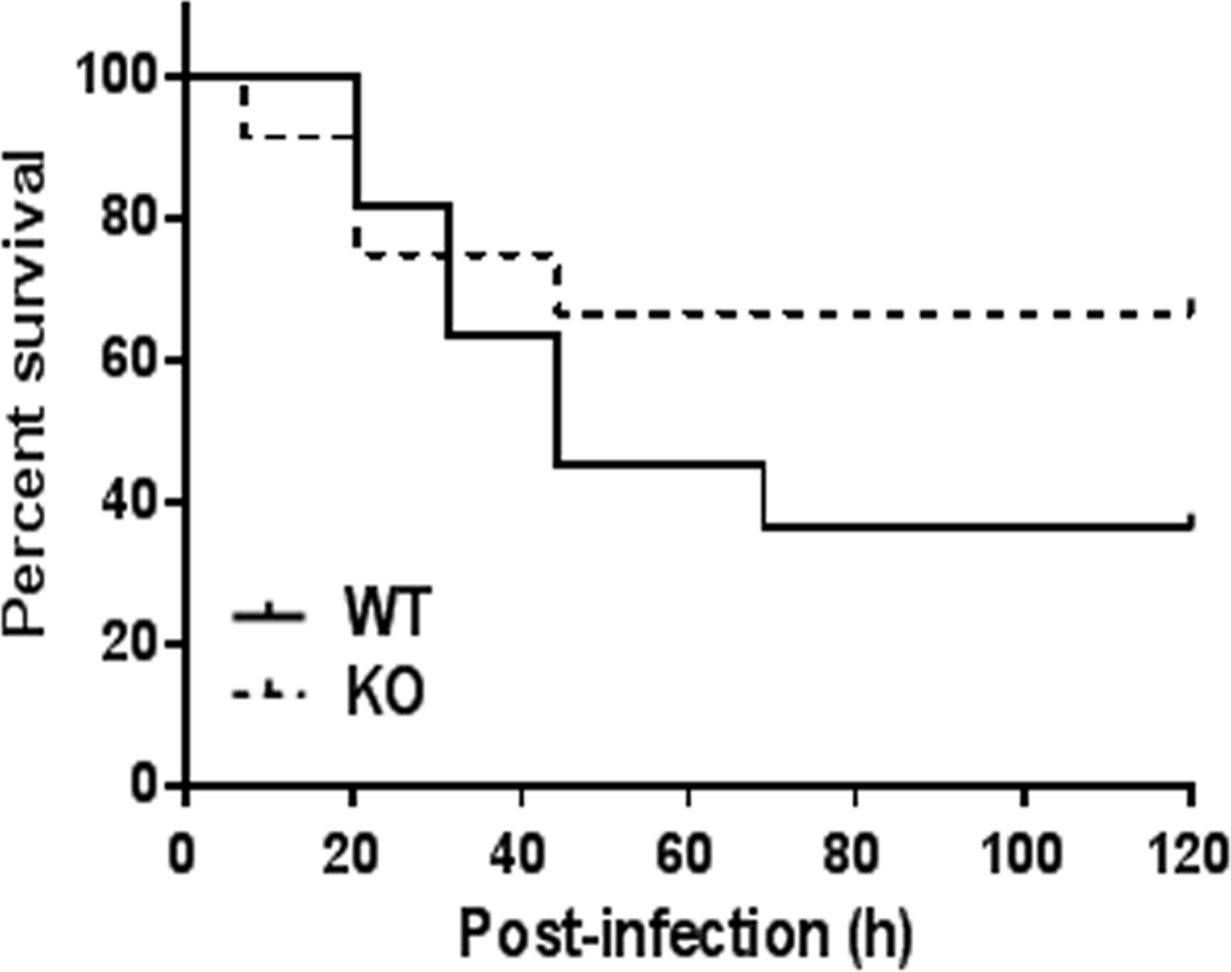
*S*. *pneumoniae* high-dose survival experiment. WT and NKLAM-KO mice were infected via intranasal route with 5 x 10^7^ *S*. *pneumoniae* CFU in 30 μL sterile PBS and monitored for signs of severe illness for 5 days. WT, n = 11; NKLAM-KO, n = 12. p = not significant.

We compared wild type and NKLAM-KO mice for their ability to survive a high dose of *S*. *pneumoniae*. Mice were nasally infected with 5 x 10^7^ CFU of *S*. *pneumoniae* and monitored for signs of distress. The survival curves indicate that 67% of NKLAM-KO mice (n = 12) survived 72 hr post-infection while only 36% of WT mice (n = 11) survived (Fig 7). The experiment was continued for 120 h post-infection with no more deaths observed in either group and both groups of surviving mice were behaving normally with no overt signs of infection.

## Discussion

*Streptococcus pneumoniae* is a Gram-positive bacterium that is the source of significant human mortality and morbidity and is a common cause of pneumonia and meningitis. We used an *in vivo* mouse model of pneumococcal infection to further examine the role of ubiquitin ligase NKLAM in host defense.

Colonization of the upper respiratory tract by *S*. *pneumoniae* induces a pro-inflammatory response initiated by Toll-like receptors (TLR) with subsequent activation of the NFκB cascade and activation of STAT proteins [22, 23]. We have shown previously that NKLAM positively affects both NFκB and STAT1-mediated transcriptional activity [8, 12]. Thus, we hypothesized that NKLAM-KO mice would have difficulty mounting an immune response against *S*. *pneumoniae*. We found that the lack of NKLAM leads to decreased killing as assessed by *in vitro* and *in vivo* assays. Both neutrophils and macrophages from NKLAM-KO mice were less effective in killing *S*. *pneumoniae* compared to WT mice. NKLAM-KO mice had over twice the amount of *S*. *pneumoniae* CFU/lung than WT mice (Fig 1) 24h-post infection. Although the difference in bacteria burden between WT and NKLAM-KO was significant but small, it does aid in solidifying the role of NKLAM as a component of the innate immune system. After 48h of infection, the CFU/lung in both genotypes were similar. This suggests that NKLAM may be involved primarily in inflammatory processes during the initial stages of infection. Another possible scenario is that there are compensatory mechanisms at the protein level that come into play in the NKLAM-KO animals. RBR family member Parkin is a good candidate for compensation during bacterial killing, as the lack of Parkin is associated with increased bacterial load and mortality in an *in vivo* tuberculosis infection model [6]. Dorfin, another RBR E3 ubiquitin ligase family member, has identical protein structure and is 46% homologous to NKLAM. Due to this similarity, it is conceivable that Dorfin may play a role in host defense similar to NKLAM. Currently, studies on the role of Dorfin in host defense are limited. However, there is evidence that Dorfin, along with other E3 ubiquitin ligases, plays a role in inhibiting HIV infectivity [24].

Cytokine expression facilitates the progression of an immune response via leukocyte differentiation, migration and activation. We have established a role for NKLAM in cytokine production at the mRNA level; thus, in our next set of experiments we assayed lung homogenate and plasma from *S*. *pneumoniae*-infected mice for cytokine levels using the cytometric bead array. Overall, we found the cytokine levels in the lung mirrored our qPCR results. Interferon gamma and MCP-1 from lung homogenates and plasma were significantly reduced in infected NKLAM-KO mice compared to WT mice (Fig 4A and 4B). The levels of TNFα were also lower in NKLAM-KO mice. The promoter region of TNFα contains STAT1, STAT3 and STAT5/6 consensus binding sites [25, 26] and TNFα expression can be induced by the JAK/STAT pathway following treatment with IFNγ [27]. Thus, diminished IFNγ production is a potential explanation for the lower TNFα expression in NKLAM-KO mice. Interleukin 12, a pro-inflammatory cytokine produced by macrophages and B cells, was reduced in *S*. *pneumoniae*-infected NKLAM-KO lung homogenates at 24 and 48h. IL-12 induces the production of IFNγ from NK cells and T cells [28] and facilitates Th1 helper cell differentiation [29]. IL-12 is composed of p35 and p40 subunits encoded by separate genes [30]. The p35 subunit is constitutively expressed and contains consensus DNA binding sites for STAT proteins [31], while the p40 subunit, which is upregulated by bacterial stimuli, contains NFκB binding sites within its promoter region [30]. Previous data from our laboratory has shown that NKLAM is involved in the nuclear translocation of NFκB and DNA binding affinity of STAT; thus, NKLAM may play a role in IL-12 expression through modulation of transcription factor activity.

Based upon the observation that NKLAM-KO mice had a significant defect in STAT1 tyrosine phosphorylation, we hypothesized that the expression of STAT1 target genes would be lower in NKLAM-KO mice. Indeed, we found that NKLAM-KO mice were unable to express several immunologically relevant STAT1 target genes to the same degree as WT mice. The expression of iNOS was lower in NKLAM-KO lungs and macrophages as compared to WT mice (Figs 5A and 3C). We also found that MCP-1 and MIP1α, two chemokines important for the recruitment of leukocytes to sites of infection [20, 32, 33], were significantly lower in NKLAM-KO mice at 24h-post infection (Fig 5B). While the levels of MCP-1 were decreased in NKLAM-KO lungs at 24 and 48h, the levels of MIP1α at 48h were significantly increased compared to the 24h timepoint yet the levels were similar between genotypes (Fig 5) suggesting that other signaling mechanisms may come into play later in infection that normalize leukocyte migration in the NKLAM-KO mice. At the protein level, RBR family member Parkin is an attractive possibility as Parkin has been shown to control, in part, neutrophil migration and activation as well as the production of inflammatory cytokines IL-6 and TNFα in BAL fluid [13].

STAT family proteins play significant roles in host defense, and our studies demonstrated an attenuation of STAT phosphorylation in infected NKLAM-KO mouse lungs; an effect that could involve kinases and/or phosphatases. Our whole lung in-gel phosphatase assay (Fig 6F) showed that NKLAM-KO mice also have enhanced total phosphatase activity at 24h-post infection. We must note that the read-out for this assay is the result of all cellular phosphatase activities within the lung; however, increased phosphatase lung activity is a plausible explanation for the reduced STAT phosphorylation. Another possible scenario is that NKLAM ubiquitinates and degrades a phosphatase that normally dephosphorylates STAT1; thus, in cells lacking NKLAM, phosphatase expression would be prolonged. Investigation into a potential NKLAM-associated phosphatase is an ongoing effort in the laboratory. Both NKLAM and Parkin have been shown to associate with phosphatases [34, 35]. Additionally, members of the Cbl, Hakai, SOCS-Cul5-RING families of ubiquitin ligases require their targets to be tyrosine phosphorylated prior to binding and ubiquitination [36]; thus, ubiquitin ligases that target tyrosine phosphoproteins could localize to signal transduction complexes and in turn regulate signal transduction via protein degradation.

The failure of NKLAM-KO mice to mount an immune response to *S*. *pneumoniae* that was comparable to WT mice turned out to be beneficial as disease severity in the NKLAM-KO was less. The lack of NKLAM protein was associated with less lung inflammation and the survival study showed a trend toward decreased mortality. This is in line with studies that show excessive pulmonary leukocyte infiltration can lead to tissue damage and acute respiratory distress [37, 38]. Future studies with organisms that provide a stronger, more prolonged inflammatory response will allow us to elucidate additional information regarding the function of NKLAM in the immune response to pathogens.

In conclusion, we provide novel data that E3 ubiquitin ligase NKLAM is a positive regulator of innate immunity. We suggest that NKLAM, through its modulation of key immunological transcription factors, regulates the expression of cytokines and chemokines that are critical for directed migration of neutrophils and NK cells into the lungs of *S*. *pneumoniae*-infected mice.

## References

[1] Center for Disease Control and Prevention. Pneumococcal Disease 2015 [updated June 10, 2015March 3. 2017]. Available from: https://www.cdc.gov/pneumococcal/clinicians/streptococcus-pneumoniae.html.

[2] Appelbaum PC. Resistance among Streptococcus pneumoniae: Implications for drug selection. Clin Infect Dis. 2002;34(12):1613–20. doi: 10.1086/340400 12032897 10.1086/340400

[3] Center for Disease Control and Prevention. Antibiotic/Antimicrobial Resisitance 2016 [March 3, 2017]. Available from: https://www.cdc.gov/drugresistance/biggest_threats.html.

[4] Eisenhaber B, Chumak N, Eisenhaber F, Hauser MT. The ring between ring fingers (RBR) protein family. Genome Biol. 2007;8(3):209 doi: 10.1186/gb-2007-8-3-209 17367545 10.1186/gb-2007-8-3-209 PMC1868946

[5] Lawrence DW, Kornbluth J. E3 ubiquitin ligase NKLAM is a macrophage phagosome protein and plays a role in bacterial killing. Cell Immunol. 2012;279(1):46–52. doi: 10.1016/j.cellimm.2012.09.004 23085241 10.1016/j.cellimm.2012.09.004 PMC3518603

[6] Manzanillo PS, Ayres JS, Watson RO, Collins AC, Souza G, Rae CS, et al The ubiquitin ligase parkin mediates resistance to intracellular pathogens. Nature. 2013;501(7468):512–6. doi: 10.1038/nature12566 24005326 10.1038/nature12566 PMC3886920

[7] Mira MT, Alcais A, Nguyen VT, Moraes MO, Di Flumeri C, Vu HT, et al Susceptibility to leprosy is associated with PARK2 and PACRG. Nature. 2004;427(6975):636–40. doi: 10.1038/nature02326 14737177 10.1038/nature02326

[8] Lawrence DW, Kornbluth J. E3 ubiquitin ligase NKLAM ubiquitinates STAT1 and positively regulates STAT1-mediated transcriptional activity. Cell Signal. 2016;28(12):1833–41. doi: 10.1016/j.cellsig.2016.08.014 27570112 10.1016/j.cellsig.2016.08.014 PMC5206800

[9] Chapgier A, Boisson-Dupuis S, Jouanguy E, Vogt G, Feinberg J, Prochnicka-Chalufour A, et al Novel STAT1 alleles in otherwise healthy patients with mycobacterial disease. PLoS Genet. 2006;2(8):e131 doi: 10.1371/journal.pgen.0020131 16934001 10.1371/journal.pgen.0020131 PMC1550284

[10] Chapgier A, Kong XF, Boisson-Dupuis S, Jouanguy E, Averbuch D, Feinberg J, et al A partial form of recessive STAT1 deficiency in humans. J Clin Invest. 2009;119(6):1502–14. doi: 10.1172/JCI37083 19436109 10.1172/JCI37083 PMC2689115

[11] Dupuis S, Jouanguy E, Al-Hajjar S, Fieschi C, Al-Mohsen IZ, Al-Jumaah S, et al Impaired response to interferon-alpha/beta and lethal viral disease in human STAT1 deficiency. Nat Genet. 2003;33(3):388–91. doi: 10.1038/ng1097 12590259 10.1038/ng1097

[12] Lawrence DW, Gullickson G, Kornbluth J. E3 ubiquitin ligase NKLAM positively regulates macrophage inducible nitric oxide synthase expression. Immunobiology. 2015;220(1):83–92. doi: 10.1016/j.imbio.2014.08.016 25182373 10.1016/j.imbio.2014.08.016 PMC4278644

[13] Letsiou E, Sammani S, Wang H, Belvitch P, Dudek SM. Parkin regulates lipopolysaccharide-induced proinflammatory responses in acute lung injury. Transl Res. 2017;181:71–82. doi: 10.1016/j.trsl.2016.09.002 27693468 10.1016/j.trsl.2016.09.002 PMC12951548

[14] de Leseleuc L, Orlova M, Cobat A, Girard M, Huong NT, Ba NN, et al PARK2 mediates interleukin 6 and monocyte chemoattractant protein 1 production by human macrophages. PLoS Negl Trop Dis. 2013;7(1):e2015 doi: 10.1371/journal.pntd.0002015 23350010 10.1371/journal.pntd.0002015 PMC3547867

[15] Inn K-S, Gack MU, Tokunaga F, Shi M, Wong L-Y, Iwai K, et al Linear Ubiquitin Assembly Complex Negatively Regulates RIG-I- and TRIM25-Mediated Type I Interferon Induction. Mol Cell. 2011;41(3):354–65. doi: 10.1016/j.molcel.2010.12.029 21292167 10.1016/j.molcel.2010.12.029 PMC3070481

[16] Hoover RG, Gullickson G, Kornbluth J. Impaired NK cytolytic activity and enhanced tumor growth in NK lytic-associated molecule-deficient mice. J Immunol. 2009;183(11):6913–21. doi: 10.4049/jimmunol.0901679 19915045 10.4049/jimmunol.0901679

[17] Swamydas M, Lionakis MS. Isolation, purification and labeling of mouse bone marrow neutrophils for functional studies and adoptive transfer experiments. J Vis Exp. 2013(77):e50586 doi: 10.3791/50586 23892876 10.3791/50586 PMC3732092

[18] Kozlowski M, Schorey J, Portis T, Grigoriev V, Kornbluth J. NK lytic-associated molecule: a novel gene selectively expressed in cells with cytolytic function. J Immunol. 1999;163(4):1775–85. 10438909

[19] Menten P, Wuyts A, Van Damme J. Macrophage inflammatory protein-1. Cytokine Growth Factor Rev. 2002;13(6):455–81. 12401480 10.1016/s1359-6101(02)00045-x

[20] Standiford TJ, Kunkel SL, Lukacs NW, Greenberger MJ, Danforth JM, Kunkel RG, et al Macrophage inflammatory protein-1 alpha mediates lung leukocyte recruitment, lung capillary leak, and early mortality in murine endotoxemia. J Immunol. 1995;155(3):1515–24. 7636213

[21] Satoh J, Tabunoki H. A Comprehensive Profile of ChIP-Seq-Based STAT1 Target Genes Suggests the Complexity of STAT1-Mediated Gene Regulatory Mechanisms. Gene regulation and systems biology. 2013;7:41–56. doi: 10.4137/GRSB.S11433 23645984 10.4137/GRSB.S11433 PMC3623615

[22] Parker D, Martin FJ, Soong G, Harfenist BS, Aguilar JL, Ratner AJ, et al Streptococcus pneumoniae DNA initiates type I interferon signaling in the respiratory tract. mBio. 2011;2(3):e00016–11. doi: 10.1128/mBio.00016-11 21586648 10.1128/mBio.00016-11 PMC3101776

[23] Amory-Rivier CF, Mohler J, Bedos JP, Azoulay-Dupuis E, Henin D, Muffat-Joly M, et al Nuclear factor-kappaB activation in mouse lung lavage cells in response to Streptococcus pneumoniae pulmonary infection. Crit Care Med. 2000;28(9):3249–56. 11008989 10.1097/00003246-200009000-00021

[24] Liu L, Oliveira NM, Cheney KM, Pade C, Dreja H, Bergin AM, et al A whole genome screen for HIV restriction factors. Retrovirology. 2011;8:94 doi: 10.1186/1742-4690-8-94 22082156 10.1186/1742-4690-8-94 PMC3228845

[25] Chappell VL, Le LX, LaGrone L, Mileski WJ. Stat proteins play a role in tumor necrosis factor alpha gene expression. Shock. 2000;14(3):400–2; discussion 2–3. 11028563 10.1097/00024382-200014030-00027

[26] Takagi K, Takagi M, Kanangat S, Warrington KJ, Shigemitsu H, Postlethwaite AE. Modulation of TNF-alpha gene expression by IFN-gamma and pamidronate in murine macrophages: regulation by STAT1-dependent pathways. J Immunol. 2005;174(4):1801–10. 15699106 10.4049/jimmunol.174.4.1801

[27] Vila-del Sol V, Punzon C, Fresno M. IFN-gamma-induced TNF-alpha expression is regulated by interferon regulatory factors 1 and 8 in mouse macrophages. J Immunol. 2008;181(7):4461–70. 18802049 10.4049/jimmunol.181.7.4461

[28] Vignali DA, Kuchroo VK. IL-12 family cytokines: immunological playmakers. Nat Immunol. 2012;13(8):722–8. doi: 10.1038/ni.2366 22814351 10.1038/ni.2366 PMC4158817

[29] Trinchieri G. Interleukin-12 and the regulation of innate resistance and adaptive immunity. Nat Rev Immunol. 2003;3(2):133–46. doi: 10.1038/nri1001 12563297 10.1038/nri1001

[30] Murphy TL, Cleveland MG, Kulesza P, Magram J, Murphy KM. Regulation of interleukin 12 p40 expression through an NF-kappa B half-site. Mol Cell Biol. 1995;15(10):5258–67. 7565674 10.1128/mcb.15.10.5258 PMC230773

[31] Collison LW, Delgoffe GM, Guy CS, Vignali KM, Chaturvedi V, Fairweather D, et al The composition and signaling of the IL-35 receptor are unconventional. Nat Immunol. 2012;13(3):290–9. doi: 10.1038/ni.2227 22306691 10.1038/ni.2227 PMC3529151

[32] Balamayooran G, Batra S, Balamayooran T, Cai S, Jeyaseelan S. Monocyte chemoattractant protein 1 regulates pulmonary host defense via neutrophil recruitment during Escherichia coli infection. Infect Immun. 2011;79(7):2567–77. doi: 10.1128/IAI.00067-11 21518788 10.1128/IAI.00067-11 PMC3191985

[33] Matsukawa A, Hogaboam CM, Lukacs NW, Lincoln PM, Strieter RM, Kunkel SL. Endogenous monocyte chemoattractant protein-1 (MCP-1) protects mice in a model of acute septic peritonitis: cross-talk between MCP-1 and leukotriene B4. J Immunol. 1999;163(11):6148–54. 10570305

[34] Fardilha M, Esteves SL, Korrodi-Gregorio L, Pelech S, da Cruz ESOA, da Cruz ESE. Protein phosphatase 1 complexes modulate sperm motility and present novel targets for male infertility. Mol Hum Reprod. 2011;17(8):466–77. doi: 10.1093/molehr/gar004 21257602 10.1093/molehr/gar004

[35] Kurup PK, Xu J, Videira RA, Ononenyi C, Baltazar G, Lombroso PJ, et al STEP61 is a substrate of the E3 ligase parkin and is upregulated in Parkinson's disease. Proc Natl Acad Sci U S A. 2015;112(4):1202–7. doi: 10.1073/pnas.1417423112 25583483 10.1073/pnas.1417423112 PMC4313846

[36] Cooper JA, Kaneko T, Li SS. Cell regulation by phosphotyrosine-targeted ubiquitin ligases. Mol Cell Biol. 2015;35(11):1886–97. doi: 10.1128/MCB.00098-15 25776560 10.1128/MCB.00098-15 PMC4420928

[37] Narayana Moorthy A, Narasaraju T, Rai P, Perumalsamy R, Tan KB, Wang S, et al In vivo and in vitro studies on the roles of neutrophil extracellular traps during secondary pneumococcal pneumonia after primary pulmonary influenza infection. Frontiers in immunology. 2013;4:56 doi: 10.3389/fimmu.2013.00056 23467809 10.3389/fimmu.2013.00056 PMC3587798

[38] Neill DR, Fernandes VE, Wisby L, Haynes AR, Ferreira DM, Laher A, et al T regulatory cells control susceptibility to invasive pneumococcal pneumonia in mice. PLoS Pathog. 2012;8(4):e1002660 doi: 10.1371/journal.ppat.1002660 22563306 10.1371/journal.ppat.1002660 PMC3334885

